# Medical News and Miscellany

**Published:** 1889-10

**Authors:** 


### MEDICAL NEWS AND MISCELLANY.

MARRIED.—At Taylor, Texas, October 10, inst., Dr. Lunsford P. Black to Miss Mollie Tucker, both of that city. We extend our congratulations to the doctor, and say to all our young friends, "Go thou and do like "Black."

---

WANTED—The present address of Mr. J. S. O. Brooks, traveling in Texas for D. Appleton & Co.'s publications; last heard from at Marshall, Texas. Any friend knowing where a letter will reach Mr. Brooks, will confer a favor by dropping us a postal card giving the information.

---

THE AUSTIN DISTRICT MEDICAL SOCIETY and the Travis County Medical Society are considering the propriety of fitting up handsome quarters for a permanent home in the capital city. It is the intention of the two societies to collect pathological specimens and properly take care of them. Also, to establish a library, the nucleus of which is now on hand. The exchanges of this JOURNAL, consisting of near a hundred periodicals monthly, have been donated.

---

PAY-DAY has arrived for "all hands and the cook," and yours truly *certainly* expects his subscribers to pay up arrears. There is no excuse for not doing so, and a man of any sensibility should be ashamed to take a journal year after year and not pay for it. Our subscribers who have been prompt have our grateful acknowledgements, and we hope, they will promptly pay for the current volume—(5)—beginning July, 1889. Small favors thankfully received, and we will remember in our prayers all who remit on receipt of this splendid issue of the JOURNAL.

---

REMOVALS.—Gr. G. B. Turner has removed from Richmond to Temple.

Dr. L. Merriwether from Waxahachie to Grapeland.

Dr. R. A. Taylor from Millwood to Nevada, same county, Collin.

Dr. W. B. Watkins, from San Angelo to Stony Point, Van Zandt county.

Dr. J. A. Armstrong, of Salado, is at the N. Y. Polyclinic, attending a special course.

---

DR. I. N. LOVE, of St. Louis, in the *New England Medical Monthly*, gives a glowing account of his trip, in company with a number of other distinguished gentlemen, to the celebrated manufacturing establishment of Carnrick's Soluble Food and Lacta Preparata. He says, Goshen is veritably a land flowing with milk—if not with honey, and he describes, in detail, the process by which these infant foods are prepared—testifying at the same time to their absolute purity and fitness for the purposes for which they are recommended, and so largely used. See Reed & Carnrick's advertisement.

### FALSE SWEARING.

FICTITIOUS COURT DECISIONS BACKED UP BY LYING AFFIDAVITS FOR THE SAKE OF SELLING A SUBSTITUTE EXTRACT.

*To the Medical Profession:*

Physicians who have been induced to prescribe the *substitute* Hoft's Malt Extract, which is put up in a *short*, *squatty* bottle, bearing the name of "Johann Hoff," and "Moritz Eisner" on the neck, and sold as the genuine or the imported Hoff's Malt Extract, will no doubt, be surprised to learn that the proprietors of that article have just been convicted in a Berlin court, of circulating a fictitious and maliciously false court decision and of swearing to a false affidavit.

The *genuine* Hoff's Malt Extract was introduced into this country in 1866 by the manufacturer, Mr. Hoff, of Hamburg, and *was sold in a short, squatty bottle.* In 1869 a new slender and handsome green bottle was adopted for the sales in this market, and the agency for the United States and the British Provinces of North America was transferred to us.

In 1880 a *new firm*, styling itself "Johann Hoff," started up in Berlin, and began *mixing* and putting on the market a *substitute Hoff's Malt Extract.* Moritz Eisner, then of Philadelphia, now of the Eisner & Mendelson Company, No. 6. Barclay street, New York, became the American agent.

The manufacturers of this *substitute* adopted the bottle which had been abandoned for this market, and there is no doubt but that this led to some confusion.

Lawsuits followed, both in this country and in Germany, and in *every instance the cases were dismissed at the cost of this new firm.* They then appealed to the Supreme Court of the German Empire. On a technicality the case was sent back to the lower court for a retrial. In May, 1887, there appeared in various trade and medical journals (and notably in the *Oil, Paint and Drug Reporter*, of New York) an article headed "Another Decision on Hoff's Malt Extract." It reflected severely upon our firm and upon Mr. Leopold Hoff. It purported to be the decision of the Supreme Court of the German Empire, and was signed "Reuling, Judge." We promptly denied the authenticity of this document, as did Mr. Leopold Hoff, whereupon the Eisner & Mendelson Company, in a letter printed in the *Oil, Paint and Drug Reporter*, of June 22, 1887, without attempting to answer our allegations concerning the spurious character of the article whose insertion was caused by them, stated that they had in their possession an exact copy of the decision of the German Supreme Court for any one's perusal, and printed in the letter the names in full of the judges who signed the genuine decision. If they had, they must have known the document which had already appeared, and which was freely circulated through the mails over a year afterwards, was fraudulent. An investigation proved the document to be a *garbled copy* of the plea of a lawyer, named Reuling, who argued the case of the firm styling itself Johann Hoff before the German Supreme Court.

In January, 1888, Max Martin Hoff, one of the partners of the so-called firm "Johann Hoff," for whom the Eisner & Mendelson Company are agents, made an affidavit before a Philadelphia notary public that their lawyer's plea, which had been published as the decision of the Supreme Court of the German Empire, was a *true* and *correct* copy of that decision, and this lying affidavat with the false court decision were printed side by side and circulated extensively among the medical men and drug trade of this country. At the time he made this affidavit he was then in Philadelphia, superintending the mixing of the substitute article on the premises of the G. Manz Brewing Company, Sixth and Clearfield streets.

Copies of these publications being sent to Mr. Leopold Hoff, he promptly sued the Berlin firm for damages and to enjoin them from their further circulation, and on June 13, 1889, the following decision was rendered, which we reproduce as cabled to us Mr. Lang, the then U. S. Consul at Hamburg, per our request:

"HAMBURG, June 28, 1889.

"Tarrant & Co., New York: As requested, I send extract translated from certified decision of the Berlin Court Prussian Royal Landsgericht, rendered June 13, 1889, in the case of Leopold Hoff vs. 'Johann Hoff':

"Defendants admit that they have sent to Eisner & Mendelson the paper filed by their lawyer, Reuling, in the Hamburg Revision case. It is not to be doubted that defendants have done it in order that the paper should be used by their agents for advertising their preparation against plaintiff or Tarrant & Co.

"The defendants say the publication was only an error of their agents (Eisner & Mendelson Co.), but it is clear that the defendants have directly ordered their agents to publish their own paper as the decision of the Reichsgericht. According to the opinion of the Court this intention existed from the beginning. Max Martin Hoff *admits* having confirmed the statement that the published decision is that of Reichsgericht, but it is proved that *he has sworn to it.* By signing the affidavit he has acted carelessly in the highest degree if he has sworn without taking notice of the contents of the paper, and it must be taken that Max Martin

Hoff swore to the paper with the intention of maintaining the publication that the same is the decision of the Reichsgericht.

"Decision as to the amount of damages is reserved.

"(Signed) LANG, U. S. Consul."

What a terrible rebuke this is to both the proprietors and the agents of this substitute extract!

What confidence can be placed in the virtue of a preparation or upon the claim of a firm convicted of circulating false court decisions and of swearing to lying affidavits?

Will the medical profession jeopardize the health of their patients by allowing them to take the *mixtures* of such unprincipled parties.

Whether they *mix* their preparation in Berlin or in Philadelphia, any physician who will take the trouble to examine it will find it to be a light, sour and nauseous liquid, bearing evidence in odor, taste and appearance of a *doctored beer*, having the effect of a stimulant, while the genuine and original Hoff's Malt, "*Tarrant's*," is a *nutrient*. In this connection we desire to say that complaints frequently reach us to the effect that "patients are nauseated and unable to retain the Malt, but upon investigation we have found in every instance that it was *the substitute Hoff's Malt* that was complained of. We have also learned that, failing to get results from using the *squatty bottle*, many physicians (supposing that to be the genuine article) have abandoned the use of Hoff's Malt.

In order to protect physicians and their patients, and draw an unmistakable line between the *original* and the substitute, the original and genuine imported preparation is now labeled

Hoff's Malt Extract, *Tarrant's*, and if, when prescribing, physicians will specify "Tarrant" no mistakes can occur. The genuine article, as stated above, is put up in a slender green bottle. It is of agreeable taste and besides being the original it is nndoubtedly the best liquid malt on the market.

It is recognized by many of our leading physicians as a standard nutritive tonic for convalescents, nursing mothers, sick children and in all wasting diseases. It is a safe, pleasant appetizer and invigorant, and was recommended by no less an authority than the late J. Milner Fothergill, M. D., as a food in typhoid fever.

Hoff's Malt, *Tarrant's*, has been imported continuously by us from Hamburg since 1869, and all that we have ever received or sold has been manufactured in the factory of Mr. Leopld Hoff, who originally introduced this preparation into the United States in 1866. TARRANT & CO., New York.

Established 1854.

